# Incidence of Tuberculosis and Associations with Indicators of Alcohol Consumption in Three Regions of Northwest Russia in 1975–2009: A Time-Series Analysis

**DOI:** 10.1155/2013/693963

**Published:** 2013-06-11

**Authors:** V. N. Kuznetsov, K. V. Shelygin, A. M. Grjibovski, A. O. Mariandyshev, E. Johansson, G. A. Bjune

**Affiliations:** ^1^Institute for Health and Society, University of Oslo, P.O. Box 1130 Blindern, 0318 Oslo, Norway; ^2^Institute of Mental Medicine, Northern State Medical University, Troitski Avenue 51, Arkhangelsk 163000, Russia; ^3^International School of Public Health, Northern State Medical University, Troitski Avenue 51, Arkhangelsk 163000, Russia; ^4^Department of International Public Health, Norwegian Institute of Public Health, P.O. Box 4404 Nydalen, 0403 Oslo, Norway; ^5^Department of Tuberculosis, Northern State Medical University, Novgorodski 163000 28, Arkhangelsk 163002, Russia; ^6^Karolinska Institute, Solnavägen 1, Solna, 171 77 Stockholm, Sweden

## Abstract

*Background*. Alcohol has several social consequences that are associated with increased risk of tuberculosis. However, there have been no studies assessing the links between tuberculosis and alcohol consumption in northwest Russia. The aim of this study was to assess associations between the incidence of tuberculosis and indicators of alcohol consumption in three regions of northwest Russia. *Methods*. The study was performed in Arkhangelsk, Murmansk and Vologda regions using the data from 1975 to 2009. Deaths from alcohol poisoning and the incidence of alcohol psychoses were used as indicators of alcohol consumption. Associations between the incidence of tuberculosis and the above mentioned indicators were studied using time-series analysis. *Results*. We identified significant positive associations between the incidence of tuberculosis and the incidence of alcohol psychoses in the same year in Arkhangelsk region (*β* = 0.24, 95% CI: 0.10–0.37) and in Vologda region (*β* = 0.18, 95% CI: 0.10–0.25), but not in Murmansk region. *Conclusions*. We found an association between the incidence of alcohol psychoses and the incidence of tuberculosis in the same year in Arkhangelsk and Vologda regions suggesting an indirect link between excessive levels of alcohol consumption and the incidence of tuberculosis in Russia.

## 1. Introduction

Russia is one of the countries with the highest incidence of tuberculosis in the world. The incidence of tuberculosis on the national level was 85.1 per 100,000 in 2008 [1]. However, a considerable reduction in the incidence of the disease has been observed in recent years, particularly in northwest Russia. For example, in the Arkhangelsk region the incidence of tuberculosis decreased from 104.0 per 100,000 in 2000 to 52.2 per 100,000 in 2011 while the overall number of deaths from tuberculosis decreased from 16.2 to 6.8 per 100,000 during the same period [2]. However, multidrug-resistant (MDR) strains of mycobacterium were found in 35.5% of new cases diagnosed in the region [3]. Most of those who are diagnosed with MDR-tuberculosis are alcohol abusers [4].

Socioeconomic factors, such as poverty, poor access to health care services, crowded housing, poor nutrition, poor general health, smoking, and alcohol abuse, have been shown to be associated with tuberculosis [5]. The associations between indicators of alcohol use and the incidence of tuberculosis are well described in the literature [6–9]. The risk of having active tuberculosis is three times as high as among persons with alcohol-related problems than among their counterparts without alcohol problems [10]. In St. Petersburg, for example, over half of patients with tuberculosis have been shown to be heavy drinkers [11].

Alcohol as a substance is not causally linked to tuberculosis. Some authors stated that high levels of alcohol consumption might be a predisposing factor for tuberculosis because of direct immunosuppressive activity of alcohol [12, 13]. Rather alcohol abuse may lead to many social problems associated with tuberculosis as well as drug resistance for antituberculosis treatment. Excessive use of alcohol often leads to employment difficulties, homelessness, social marginalization, risk of infection, reinfection, and coinfection with HIV associated with specific social mixing patterns [9–11, 14, 15]. Alcohol-related factors have been shown to be associated with treatment delay and dropout from treatment [16, 17]. Introduction of alcohol abuse monitoring into tuberculosis control and treatment system will help to identify the target group which requires specific care to reduce diagnostic delay and treatment interruptions [18, 19].

Russia has gone through major social changes in the last decades. Gorbachev's alcohol campaign started in 1985 and was accompanied by a considerable increase in life expectancy [20, 21]. The subsequent collapse of the Soviet Union in 1991 led to a significant decrease of life expectancy, particularly among men. This was followed by a partial recovery until the next economic crisis in 1998 [21, 22]. In 2004, life expectancy slowly began to increase again [22]. Many researchers linked these fluctuations in Russian life expectancy to excessive alcohol consumption. 

No studies linking tuberculosis with alcohol consumption based on long time series of the Russian data have been published internationally. The aim of this study was to assess associations between the incidence of tuberculosis and indicators of alcohol consumption in three regions of Northwest Russia.

## 2. Methods

### 2.1. Study Design and Setting

**Figure 1:**
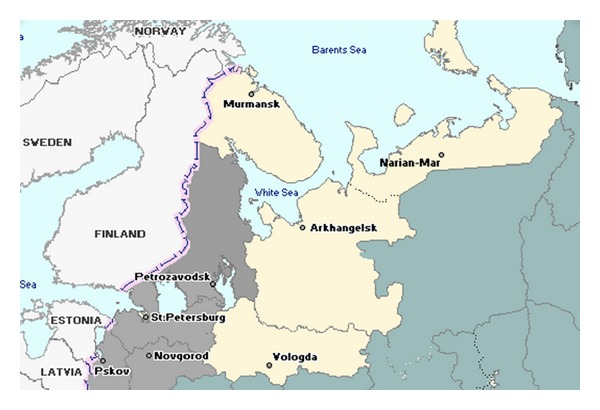
The map of Northern part of Russia.

This ecological study was performed in three regions of Northwest Russia: Arkhangelsk, Murmansk, and Vologda regions (Figure 1). The total population of these three regions was 3.3 million in 2010. 

### 2.2. Data

Alcohol poisoning deaths and incidence of alcohol psychosis were used as indicators of alcohol consumption [23]. The data on the incidence of pulmonary tuberculosis, cases of alcohol poisoning deaths, and alcohol psychoses from 1975 to 2009 were collected from the regional bureaus of statistics (Arkhangelskstat, Murmanskstat, and Vologdastat). The data were standardized by age and sex (European standard population) to ensure comparability of the results. All the data were presented separately for each region [23].

First, we presented the overall incidence of tuberculosis, but for the analysis of associations between tuberculosis and indicators of alcohol consumption we excluded cases of tuberculosis in the penitentiary system because of different patterns of both tuberculosis and alcohol consumption in prisons compared to the general population.

### 2.3. Data Analysis

To assess the association between indicators of alcohol consumption and the incidence of tuberculosis, we used autoregressive integrated moving average (ARIMA) models [24, 25]. Stationarity of the models' residuals was tested using Leung-Box Q-test, autocorrelation and partial autocorrelation functions. Model fit was assessed by maximum likelihood test. 

Deaths from alcohol poisoning and the incidence of alcohol psychoses were introduced into ARIMA models as independent variables separately. Time-series analyses were performed using SPSS 18.0 software (SPSS Inc., Chicago, IL, USA). 

## 3. Results

**Figure 2:**
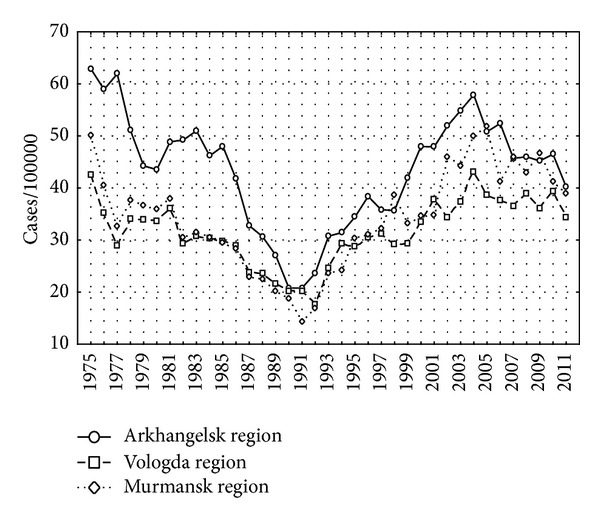
Incidence of tuberculosis per 100,000 in Arkhangelsk, Murmansk, and Vologda regions, 1975–2009.

The incidence of tuberculosis in the three regions had similar patterns during the study period (Figure 2). The incidence of tuberculosis decreased until 1991 and increased afterwards. An upward trend was reversed again in the 2000s. 

**Figure 3:**
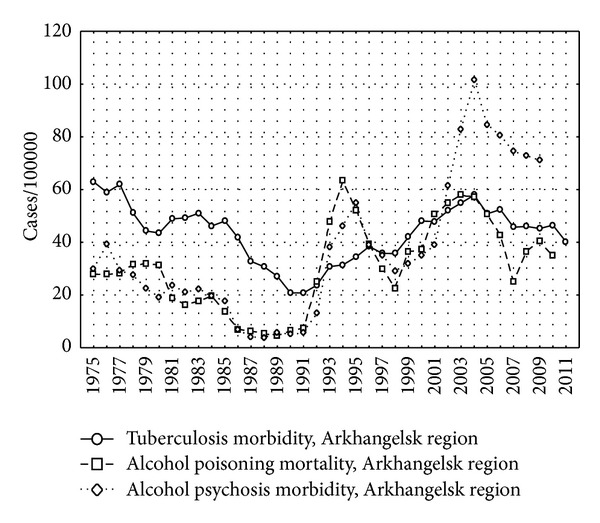
The incidence of tuberculosis, alcohol psychosis, and alcohol poisoning death per 100,000 in Arkhangelsk region, 1975–2009.

**Figure 4:**
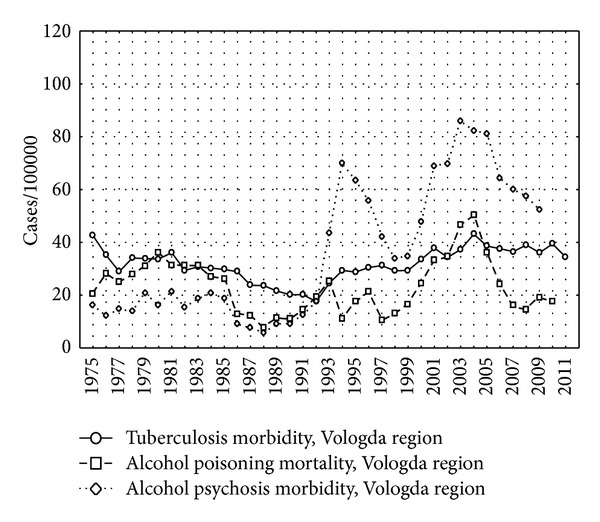
The incidence of tuberculosis, alcohol psychosis, and alcohol poisoning death per 100,000 in Vologda region, 1975–2009.

**Figure 5:**
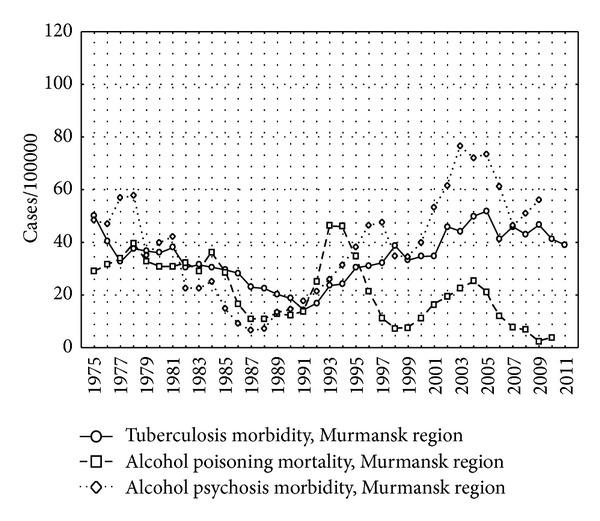
The incidence of tuberculosis, alcohol psychosis, and alcohol poisoning death per 100,000 in Murmansk region, 1975–2009.

The incidence of tuberculosis without the cases from the penitentiary system is presented separately for each region in Figures 3, 4, and 5 as well as the indicators of alcohol consumption. The peaks of alcohol poisoning death and alcohol psychosis were observed in 1993-1994 and 2003–2005, but the highest incidence of tuberculosis was registered in 2000-2001.

The results of the ARIMA models are presented in Table 1. Ljung-Box tests, autocorrelation and partial autocorrelation functions of the residuals showed acceptable model fit (data not shown). The incidence of alcohol psychosis was positively associated with the incidence of tuberculosis in Arkhangelsk and Vologda regions, but not in Murmansk region (Table 1). No associations between deaths from alcohol poisoning and the incidence of tuberculosis in any of the three regions were observed (Table 1).

## 4. Discussion

Our results suggest positive association between the incidence of alcohol psychoses and the incidence of tuberculosis in the same year in Arkhangelsk and Vologda regions, but not in Murmansk region. Our indicators of alcohol consumption have been shown to correlate well with absolute levels of alcohol consumption allowing speculations on the positive associations between alcohol consumption and the incidence of tuberculosis [26].

It is reasonable that alcohol psychoses and tuberculosis incidence may be considered as consequences of excessive chronic alcohol consumption. It was not possible to measure directly alcohol consumption in this study, so we used other indicators of alcohol consumption such as alcohol psychoses and deaths from alcohol psychosis. At the same time, alcohol poisonings are relatively common in Russia and occur in various social groups, while most of alcohol psychoses occur in marginalized groups [27]. Different studies have shown that only 3–25% of persons with alcoholism experience psychosis [28, 29]. 

These marginal groups of people are not specified in different social statistics (like jobless persons, etc.). These people are more vulnerable to tuberculosis as well. Perhaps, preventive measures aimed at decreasing of alcohol consumption in this group may lead to a decrease in the incidence of tuberculosis. 

Excessive alcohol use has social consequences that are closely correlated with risk factors for tuberculosis. Moreover, some studies have described a delay in demographic effects of antialcohol campaign, such as number of deaths, and cardiovascular diseases occurred several years late after the campaign was terminated [27]. 

According to our data, there were parallel trends of tuberculosis incidence for these three regions (Figures 2–5). The incidence had been gradually decreasing until 1991 possibly because of Gorbachev's alcohol campaign that led to increase in life expectancy [20, 21]. Then, a reverse process started after a breakup of the Soviet Union. Deterioration of the standards of living, impoverishment of the majority of the population, and reduction of preventive activities by health care system may have contributed to the reversal of trend of tuberculosis incidence. The social system was destroyed that led to discontinuance of effective TB programs [18, 30]. 

During 1995–1998, alcohol consumption in Russia was decreasing. It is shown by the level of alcohol poisoning mortality, that deaths from pancreatitis and liver cirrhosis, alcohol psychosis, and homicides started decreasing during those years [27]. According to Nemtsov, limitation of availability of alcohol beverages was the main reason for this decrease [27]. Alcohol prices increased and became relatively expensive in comparison to food [27]. We can see significant initial decreasing tuberculosis incidence in 1991. 

The peak of economical crisis of August 1998 in Russia decreased living standards for many people. Tuberculosis incidence started to increase dramatically (Figure 2). 

There was a lack of association between incidence of tuberculosis and alcohol psychosis in Murmansk region possibly by several reasons. First, lower levels of alcohol consumption in Murmansk region in comparison with Arkhangelsk and Vologda regions (Figures 3–5) may be the reason for the lack of the association. Moreover, smaller population of Murmansk region than in Arkhangelsk and Vologda regions is associated with lower statistical power, although point estimates are not similar to those obtained in the other two regions. 

Many authors described a strong association between heavy alcohol use (or alcohol use disorders) and tuberculosis (relative risk fluctuated from 2.94 to 8.58) in different countries [9–11, 31]. According to Shin et al., 23% of women and 70.6% of men had lifetime alcohol abuse or dependence among tuberculosis patients in Tomsk, Russia [32]. Substance abuse was the most commonly reported behavioral risk factor among patients with tuberculosis in the United States [33] and in the United Kingdom [34]. 

Alcohol-abusing people show social marginalization and drift that would constitute risk factors for high exposure of infection [9, 35]. Alcohol consumption very often leads to depression that associates with reduction of social level and immune suppression [36].

Most of the studies that showed associations between tuberculosis and alcohol consumption were from studies of limited groups (e.g., prisoners, local population, or patients with tuberculosis). Such systematic errors may lead to incomplete views about association of alcohol consumption with tuberculosis for a general population and may lead to false conclusion [10]. 

The strength of our research is using data of 35-year period of three regions of Northwest Russia that allows having long enough time series to study associations between alcohol and tuberculosis on a population level.

The limitation of our study is the ecological research design. Thus, the observed associations may suffer from ecological fallacy and may not indicate causal links between the exposure and the outcome on the individual level. 

## 5. Conclusion

We observed a significant association between the incidence of alcohol psychoses and the incidence of tuberculosis on a population level in the Arkhangelsk and Vologda regions, but not in Murmansk region. However, given the ecological nature of the data and potential limitations of the study, the results should be interpreted with due caution. 

## Figures and Tables

**Table 1:** Estimated parameters for ARIMA model to forecast incidence of tuberculosis for alcohol psychosis and for alcohol poisoning deaths in Arkhangelsk, Vologda, and Murmansk regions, 1975–2009.

	Coefficient	SE (95% CI)
ARIMA models for alcohol psychosis*		
TB Arkhangelsk region, Model 1 ARIMA (0,1, 0)	.24	.09 (0.10–0.37)
TB Vologda region, Model 2 ARIMA (0,1, 0)	.18	.04 (0.10–0.25)
TB Murmansk region, Model 3 ARIMA (0,1, 0)	−.10	.83 (−1.74–1.53)
ARIMA models for alcohol poisoning deaths*		
TB Arkhangelsk region, Model 1 ARIMA (0,1, 0)	−.52	.80 (−2.1–1.0)
TB Vologda region, Model 2 ARIMA (0,1, 0)	−.19	.61 (−1.72–1.0)
TB Murmansk region, Model 3 ARIMA (0,1, 0)	−.10	.83 (−1.74–1.5)

*0, 1, and 0 means *p* = 0, *d* = 1, and *q* = 0, where *p* is the number of autoregressive terms, *d* is the number of nonseasonal differences, and *q* is the number of lagged forecast errors in the prediction equation.
